# CAR T-Cell Immunotherapy Treating T-ALL: Challenges and Opportunities

**DOI:** 10.3390/vaccines11010165

**Published:** 2023-01-12

**Authors:** Anqi Ren, Xiqin Tong, Na Xu, Tongcun Zhang, Fuling Zhou, Haichuan Zhu

**Affiliations:** 1 Institute of Biology and Medicine, College of Life and Health Sciences, Wuhan University of Science and Technology, Wuhan 430081, China; 2 Department of Hematology, Zhongnan Hospital of Wuhan University, Wuhan 430071, China; 3 Key Lab of Industrial Fermentation Microbiology of the Ministry of Education & Tianjin Key Lab of Industrial Microbiology, College of Biotechnology, Tianjin University of Science and Technology, Tianjin 300457, China

**Keywords:** immunotherapy, T-ALL, CAR T, fratricide, T-cell aplasia, product contamination

## Abstract

T-cell acute lymphoblastic leukemia (T-ALL), a form of T-cell malignancy, is a typically aggressive hematological malignancy with high rates of disease relapse and a poor prognosis. Current guidelines do not recommend any specific treatments for these patients, and only allogeneic stem cell transplant, which is associated with potential risks and toxicities, is a curative therapy. Recent clinical trials showed that immunotherapies, including monoclonal antibodies, checkpoint inhibitors, and CAR T therapies, are successful in treating hematologic malignancies. CAR T cells, which specifically target the B-cell surface antigen CD19, have demonstrated remarkable efficacy in the treatment of B-cell acute leukemia, and some progress has been made in the treatment of other hematologic malignancies. However, the development of CAR T-cell immunotherapy targeting T-cell malignancies appears more challenging due to the potential risks of fratricide, T-cell aplasia, immunosuppression, and product contamination. In this review, we discuss the current status of and challenges related to CAR T-cell immunotherapy for T-ALL and review potential strategies to overcome these limitations.

## 1. Introduction

T-cell acute lymphoblastic leukemia (T-ALL) is considered to be a disease derived from the uncontrolled proliferation of mature or immature T cells [1]. Chemotherapy, targeted therapy, immunotherapy, radiation, stem cell transplantation, and surgery are presently the standard lymphoma treatments [2,3]. Treatment options for different types of T-cell lymphomas are expanding as new therapies are identified and existing therapies are improved [4,5,6]. Moreover, many novel therapies are being investigated but have not yet been approved for direct clinical treatment of patients with T-cell leukemias, particularly T-ALL. Current treatments with intensive chemotherapy protocols and allogeneic bone marrow transplantation have demonstrated a cure rate of 75% in pediatric patients and 50% in adults with T-ALL [7,8]. In particular, the subtype of early and mature T-ALL has a high relapse rate (60–74%), which makes it imperative to explore other approaches to improve antitumor activity in T-cell leukemia patients [9].

Immunotherapy including monoclonal antibodies, checkpoint inhibitors, and CAR T-cell therapy has revolutionized the treatment landscape for various cancers, including hematologic malignancies and solid tumors [10,11]. However, the aforementioned breakthroughs have not yielded clinically favorable outcomes in T-ALL.

Antibodies targeting cell surface receptors have become a promising therapy for many cancers [12,13]. IL-7Rα was found to be upregulated in the cells of relapsed patients, and a good efficacy was observed in anti-IL-7Rα monoclonal antibodies in relapsed T-ALL patient-derived xenografts (PDXs) [14]. Furthermore, Akkapeddi et al. showed that a fully human anti-IL-7Rα antibody, B12, significantly promoted T-ALL cell death in vitro and delayed tumor development in vivo [15]. However, IL-7Rα is also expressed on normal T cells, causing T-cell depletion, which has impeded applications for the clinical study of this antibody. One group initiated a phase 1 study to identify a CD3-CD38 bispecific antibody (XmAb18968) for the treatment of patients with relapsed/refractory T-ALL and the efficacy is currently under evaluation [16].

Anti-PD-1 or anti-PD-L1 antibodies have achieved remarkable success in the treatment of solid tumors [17,18,19,20] and Hodgkin’s lymphoma [21,22]. However, as malignant T cells also express PD-1 or PD-L1, checkpoint inhibitors likely block the PD-1 pathway in T-cell malignancy and could activate tumor progression [23,24,25]. For example, a recent study showed that three T-ALL patients experienced rapid progression of disease after a single dose of nivolumab, which is a PD-1 inhibitor (NCT02631746) [26], and similar results were also reported in previous studies [27]. Thus, further studies need to be conducted to reveal the mechanism of immunotherapy and assess the clinical effect of checkpoint inhibitors in T-cell malignancies. 

**Figure 1:**
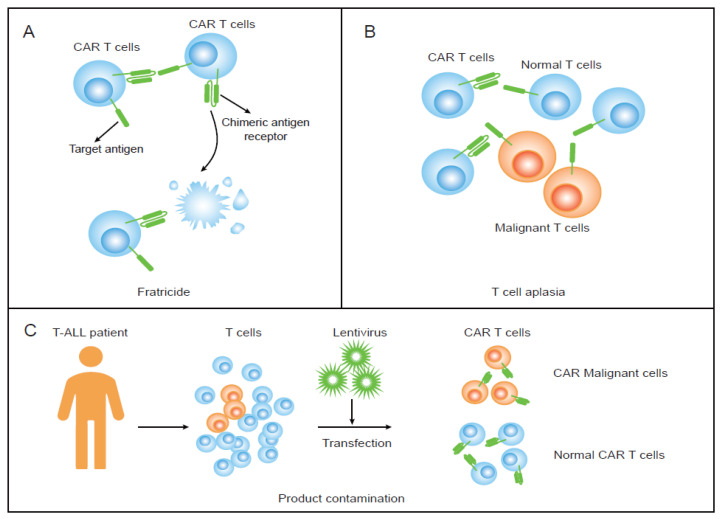
Three major challenges of CAR T therapy. (**A**) Fratricide: CAR T cells, as they have target antigens, kill each other. (**B**) T-cell aplasia: CAR T cells recognize the antigen, which has expressions on both malignant and normal T cells, and then kill the normal T cells. (**C**). Product contamination: tumor T cells are mixed in with sorted T cells and are transduced with CAR.

**Figure 2:**
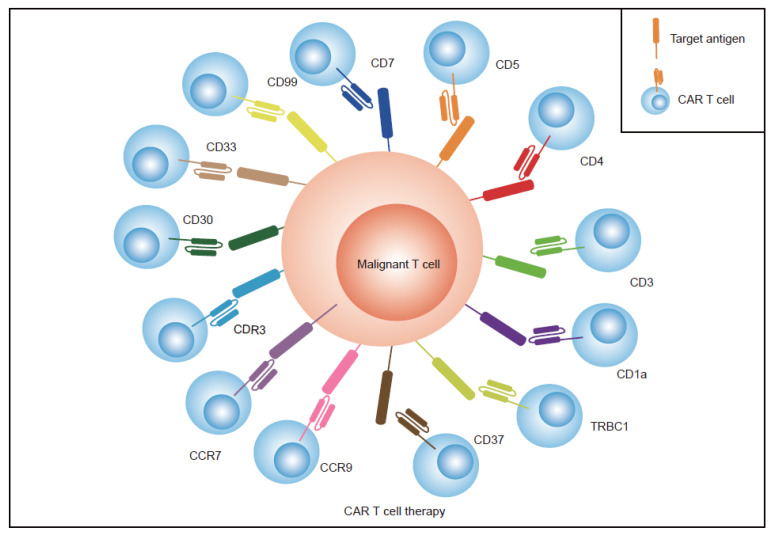
Different antigens expressed on T-cell malignancies and related targeted immunotherapies. Major CAR T-cell therapies for T-cell malignancies are displayed. Each scFv on CAR T cells is the same color as the corresponding antigen expressed on hematologic malignant cells.

Chimeric antigen receptor T-cell (CAR T) therapy has achieved great success in relapsed/refractory (R/R) hematologic malignancies [28,29,30]. The FDA has approved six CAR T products targeting CD19 for the treatment of R/R diffuse large B-cell lymphoma (DLBCL) or mantle cell lymphoma (MCL), and two targeting the B-cell maturation antigen (BCMA) for the treatment of multiple myeloma (MM) [28,31]. However, the loss of specific antigens, cell fratricide, T-cell aplasia, and tumor cell contamination are challenges in treating T-cell leukemia with CAR T therapy (Figure 1A–C) [32,33]. Cancer cells that share the same antigen as sorted T cells in vitro and normal T cells in vivo can cause CAR T cells to attack each other and normal T cells, resulting in CAR T-cell fratricide and T-cell aplasia. Furthermore, if tumor cells are mixed and transduced into CAR tumor T cells during the T-cell sorting process in CAR T cell preparation, product contamination occurs, and CAR tumor T cells proliferate in vivo, resulting in serious negative consequences. Several leukemia specifical targets (Figure 2) and different strategies are under investigation to address these problems [34,35,36,37]. Herein, we review the current investigations into CAR T immunotherapy for T-cell leukemia, including the challenges and opportunities in this field.

## 2. Fratricide and Promising Strategies

Fratricide, which is caused by the same antigen being expressed on both leukemia cells and CAR T cells, is a core problem in the development of therapies for T-cell leukemia/lymphoma, especially for T-ALL (Figure 1A). Since T-ALL is derived from normal T cells, it retains a very similar antigen expression as normal T cells, including CD7 and CD5 [38,39,40,41]. This shared expression of antigens can make CAR T cells commit fratricide, limiting the proliferation of CAR T cells in vitro and impeding cell infusion for patients. To inhibit antigen-driven fratricide, several different strategies based on the cell type or antigen have been investigated.

### 2.1. Transduce CAR beyond T Cells

Because most antigens targeted for T-cell malignancies are not shared with NK cells, and NK cells also exhibit rapid and strong cytotoxicity against tumor cells [42,43], NK cells can be adapted as allogenic CAR-modified cells to overcome the difficulty of autologous CAR T production, without the risk of GvHD given their MHC-independent activation, especially for aggressive T-ALL at relapse [44,45]. Several preclinical studies have been conducted using CAR-modified NK cells or NK-92 cell lines for the treatment of T-cell malignancies, which showed specific cytotoxicity and prolonged survival in vitro in mouse models [46,47]. Two groups reported that a specific CAR NK framework containing 2B4 (CD244) to target CD3, CD4, and CD5 had superior antitumor activity compared with the CAR T framework on NK effector cells in T-cell malignancies [48,49]. Furthermore, anti-CD7 CARs have been developed to treat T-cell malignancies with promising preclinical results, whereas effector T cells modified with anti-CD7 CARs caused extensive fratricide and prevented T-cell expansion [41,50]. Several clinical trials using CAR NK cells instead of CAR T cells for the treatment of CD7-positive T-ALL and T-cell lymphoma have been established (NCT04934774, NCT04840875) [42].

However, NK cells are difficult to expand ex vivo and transduce with viral vectors. NK-92-based CAR products could be developed as a targeted allogeneic cell therapeutic agent. This therapy requires irradiation before infusion into the patient to prevent permanent engraftment [51]. Pinz et al. constructed a CD4 CAR on NK-92 cell lines that specifically eliminated diverse CD4^+^ human T-cell leukemia/lymphoma cell lines and patient samples ex vivo and efficiently targeted and killed KARPAS-299 cells in vivo [52]. In addition to replacing NK cells with NK-92 cell lines, several methods have been developed to enhance NK cell expansion ex vivo. Kweon et al. created a new effective approach for the ex vivo expansion of human NK cells using K562 cells that were genetically engineered (GE) to express the OX40 ligand (K562-OX40L) in conjunction with brief exposure to soluble IL-21, which enhanced NK cell expansion approximately 2000-fold after 4 weeks of culture [53]. Therefore, we believe that CAR NK cell immunotherapy is a potential new avenue for the treatment of T-cell malignancies.

**Figure 3:**
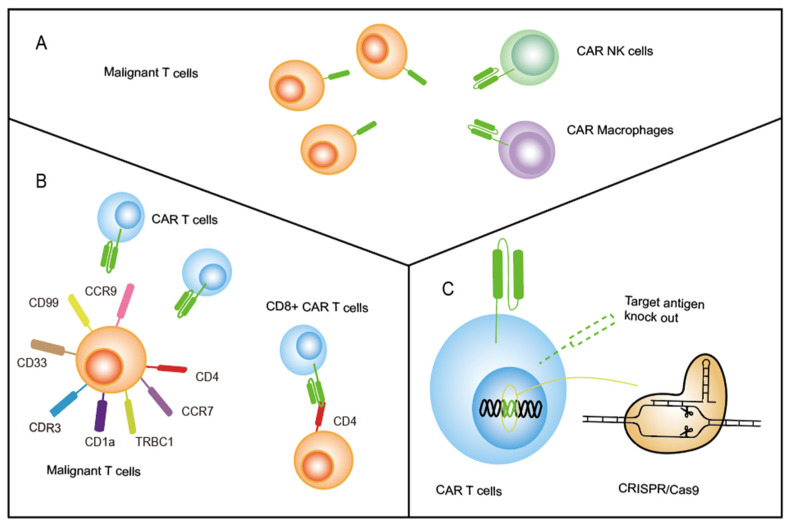
Promising strategies for CAR T-cell fratricide. (**A**) Other effector cells, such as NK cells and macrophages, replace T cells. (**B**) Identified specific antigens, with no expression in CAR T cells. (**C**) CRISPR/Cas9 knocks out the target antigen on CAR T cells.

Furthermore, CAR macrophages have emerged as an alternative therapy and have been successfully used to treat malignant tumors in vitro and in vivo, especially for solid tumors [54,55]. Various clinical trials are ongoing to determine the antitumor activity of CAR-macrophages in breast cancer, bladder cancer, lung cancer, etc. (NCT05007379, NCT04660929) [54,56]. The above results demonstrate that CAR-NK/macrophage technology is ready for clinical trials and has exhibited antitumor efficacy in T-ALL patients (Figure 3A).

### 2.2. Find Specific Antigens Restricted Expression on T Cells

Identifying tumor-specific antigens that restrict T-cell expression is another strategy to avert fratricide and overcome CAR T therapy for T-ALL (Figure 3B). Currently, TRBC1/2, CDR3, CD1a, CD4, CCR7, CCR9, CD33, CD30, and CD99 are now being identified and evaluated as targets for CAR T therapy to treat T-cell malignancies (Figure 3B, Table 1) [46,57,58,59,60,61,62,63]. These biomarkers have limited expression on normal T cells and antigen-derived CAR T cells continue to proliferate normally.

Normal T cells express either TRBC1 or TRBC2, which is coded by the T-cell receptor β-chain constant region [64]. According to normal mature T cells containing TRBC1 and TRBC2, malignancies are restricted to only one positive subtype [65]. Maciocia et al. developed anti-TRBC1 CAR T cells that recognized TCBR1-positive malignant and normal T cells but did not kill TRBC2-positive normal T cells [65]. TRBC-targeted immunotherapy was shown to eradicate a fraction of TCBR1-positive T-ALL while preserving sufficient normal T cells to maintain cellular immunity. Additionally, an ongoing clinical trial is examining the safety and efficacy of TRBC1 CAR T-cell therapy for patients with relapsed/refractory TRBC1 positive T-cell hematological malignancies (NCT04828174).

In addition, each patient has a unique T-cell receptor (TCR) on their leukemia cell surface, which could distinguish these cells from normal T cells. CDR3, the complementarity determining region 3 on TCRs, is very variable and has been used as a target for vaccines to induce antibody response [46]. Huang et al. chose unique CDR3 regions from patients with T-cell leukemia and validated the efficacy and safety of clonal T-cell CDR3-selective CAR T therapy in vitro and in vivo [46]. The CDR3-selective CAR T cells exhibited lower on-target off-tumor effects and minimized the impact on CAR T-cell amplification [46].

CD33, which is expressed on myeloid cells, is commonly used as a target of immunotherapy for AML [66,67]. Moreover, CD33 is also expressed abnormally in 10–20% of B- and T-lymphoblastic leukemias/lymphomas but not in the normal T cells. It is especially highly expressed in the subtype of early T precursor acute lymphoblastic leukemias/lymphoma (ETP-ALL) which is associated with high rates of intrinsic treatment resistance [68,69]. Guo et al. reported that CD33 was expressed in T-ALL and that CD33 highly expressed patients had a poor prognosis [70]. These results suggest that CD33 is a potential target for immunotherapy in ETP-ALL patients. However, there were not sufficient data to support the premise that targeting CD33 therapy was efficient in non-ETP T-ALL.

CCR9, a G-protein-coupled receptor for the natural ligand CCL25, is expressed on less than 5% of normal circulating T cells. Maciocia, P.M. et al. constructed anti-CCR9 CAR T cells, using a novel rat-derived anti-CCR9 scFv, and demonstrated that CCR9 CAR cells efficiently eliminated CCR9 positive tumor cells with no fratricide in vitro and in vivo [71].

Furthermore, our group also tried to identify tumor-specific antigens for T-ALL CAR T therapy and demonstrated that CD30 and CD99 are potential options for R/R T-ALL cases [59,72]. In detail, CD99 is expressed at very high levels in newly diagnosed T-ALL patients and at low levels in normal hematopoietic cells. We carried out therapy with CAR targeting CD99, which has 12E7 scFv, and achieved a significant tumor-killing effect without cytotoxicity to normal cells [59]. Two investigator-sponsored multi-institutional phase 1 studies have been established to evaluate the efficiency and safety of anti-CD99 CAR T cells based on our CAR T products, and the early clinical results are encouraging (ChiCTR2100046764, ChiCTR2000033989). 

Additionally, FACS analysis of T-ALL cell lines showed that CD21 existed on 70% of human T-ALL cell lines, and CD21 positivity varied by maturational stage, with cortical T-ALL exhibiting the highest expression (80% of cases), followed by pre-T (72%), mature (67%), ETP (25%), and pro-T (17%), suggesting that CD21 is a potential target of T-ALL [73]. Maciocia, N.C. et al. reported that CD21, which exhibits minimal expression on mature T cells, is a novel target for CAR T-cell therapy in T-ALL [73]. They successfully demonstrated the efficient treatment of anti-CD21 CAR in murine models of T-ALL.

The antigens mentioned above essentially solve the problems related to fratricide in CAR T immunotherapy. However, CAR T therapy targeting these antigens has not been widely assessed in clinical trials and these antigen-positive hematologic tumors may not cover all T-cell malignancies. More specific antigens remain to be identified.

### 2.3. Knock-Out Pan-T-Cell Targeting Antigens on CAR T Cells by CRISPR-Cas9

Genome editing techniques including TALEN, zinc-finger nucleases (ZFNs), and CRISPR/Cas9 are used to precisely modify DNA within a cell. In 2018, Rasaiyaah et al. used transcription activator-like effector nuclease (TALEN) mRNA to disrupt the TRAC locus, showing robust cytotoxicity of anti-CD3 CAR T cells against CD3-positive cells [74]. Similarly, CRISPR/Cas9 has been adapted to disrupt endogenous genes in CAR T therapy to prevent graft-versus-host disease (GvHD) [75]. Furthermore, another strategy for preventing fratricide is using CRISPR-Cas9 gene editing technology to knock out the Pan-T-cell surface antigen expressed on CAR T cells, such as CD3, CD5, and CD7, which are also present in healthy T cells (Figure 3C) [37,43,50]. For example, a preclinical study demonstrated that gene editing of CD5 in effector CAR T cells using CRISPR-Cas9 gene editing technology significantly increased the expression of CAR and the antitumor activity of anti-CD5 CAR T cells [43]. CD7, as a CAR target for T-ALL, was shown to exhibit extensive fratricide. Thus, several groups used the CRISPR/Cas9 system to disrupt the CD7 locus and demonstrated that anti-CD7 CAR T cells retained antitumor activity without killing gene-disrupted T cells in preclinical and clinical studies (NCT04264078 and NCT04004637). Recently, Hu et al. reported encouraging results from a phase 1 clinical trial (NCT04538599) in which 11 patients with refractory/relapse T-cell leukemia/lymphoma and one with CD7 positive acute myeloid leukemia received treatment of genetically modified CD7-targeting allogeneic CAR T cells. In this study, 81.8% of patients (9/11, one lost due to the efficacy evaluation) exhibited objective responses, and 63.6% (7/11, including the patient with AML) at 28 days postinfusion showed complete response [76]. In this trial, cell amplification was normal, and no dose-limiting toxicity, GvHD, immune effector cell-associated neurotoxicity, or severe cytokine release syndrome (grade  ≥  3) was observed [76]. Additionally, CRISPR/Cas9-based CD5 and CD7 gene-knockout-derived CD5/CD7 bispecific CAR T cells exhibited potent antitumor activity in vitro and in vivo [77]. 

CRISPR/Cas9-mediated editing of CAR T cells could be used to overcome CAR T-cell fratricide by targeting the antigens expressed on them. However, CRISPR/cas9 may cause DNA double-stranded breaks (DSBs), resulting in other genes being knocked out, with potentially unforeseen consequences, such as complex genomic rearrangements, megabase-scale deletions, and chromothripsis [78,79]. Recently, Diorio et al. created cytosine base editor (CBE) technology, which has more than 90% efficiency in terms of gene silencing due to point mutation without inducing DNA double-stranded breaks. She applied CBEs to create CD7-directed allogeneic CAR T using four simultaneous base edits. The CBE-edited CAR T cells exhibited normal proliferation and were shown to be highly efficient for T-ALL treatment in vitro and in vivo [80]. Thus, we believe that CAR T editing technologies are a powerful tool for the future. 

Other non-gene editing strategies have been explored to overcome CAR T fratricide. For instance, Mamonkin et al. designed a Tet-OFF expression system in which CAR expression controlled by doxycycline was imported to halt fratricide among CD5-41BB -CAR T cells in vitro and in vivo [79]. Furthermore, Png et al. used protein expression blockers (PEBLs) to maintain the targeted protein in the endoplasmic reticulum (ER)/golgi, which stopped the antigen expressing on the cell surface of CAR T cells by combining an scFv with a retention peptide. This exhibited progressively increased cytotoxicity against T-ALL blasts in vitro and significant survival advantages in vivo compared with the control group [41]. Similarly, anchoring CD7 in the ER and/or golgi to overcome CAR T fratricide was assessed in a clinical study in which all three patients were controlled (NCT04004637) [81]. In addition, Pan et al. applied donor-derived CD7 CAR T cells in a phase 1 trial for 20 r/r T-ALL patients (NCT04689659, ChiCTR2000034762) [82]. They designed CD7 CAR T without surface expression of CD7 using IntraBlock technology and reserved intracellular expression by constructing the CD7 binding domain in the vector, which could be fused with an ER retention signal sequence (KDEL) [83]. The trial achieved desirable results in which 18 patients exhibited complete remission with reversible adverse events, despite a patient dying through pulmonary hemorrhage related to fungal pneumonia after 5.5 months of transfusion. In this study, the patients’ CD7 positive T cells were killed and negative CD7 T cells exhibited normal proliferation, suggesting lower therapy-related T cell immunodeficiency [82]. Moreover, Li et al. engineered several VIPER CAR circuits, which used versatile protease to regulate CARs, and demonstrated their superior performance compared with other drug-gated systems. These results show that designing the switch to express CAR on T cells to prevent fratricide is feasible. We believe that an increasing number of technologies will be created using pan-T-cell transformation to attenuate the adverse events related to CAR T therapy.

## 3. T-Cell Aplasia and Proposed Solutions

T-cell aplasia, which is caused by on-target off-tumor toxicity of CAR T cells against normal T cells with the expression of CAR-specific target antigens, is another major obstacle to the development of CAR T cells for T-cell malignancies [33]. CAR T cells directed against such targets, which are generally expressed in normal T cells, lead to profound life-threatening infections. However, several strategies have been found to avoid the incidence of T-cell aplasia. 

**Figure 4:**
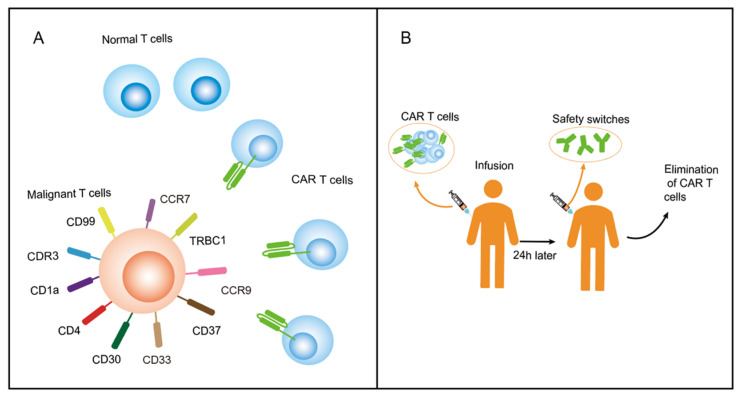
Proposed solutions of T-cell aplasia. (**A**). Select a specific antigen of malignant T cells, which has no expression or limited expression on normal T cells. (**B**). Incorporate switches into the CAR constructs to kill the CAR T cells after they perform the antitumor function.

Selecting the specific antigen of malignant T cells (no expression or limited expression on normal T cells) is an alternative method with which to circumvent the issues related to T-cell aplasia (Figure 4A). As mentioned above, several potential target antigens have been investigated, including TRBC1/2, CDR3, CD1a, CD4, CCR7, CCR9, CD30, CD33, and CD99 [46,57,58,59,61,62,84,85] (Table 1). CD1a is expressed in cortical T-ALL, which comprises 35–40% of all T-ALL cases [66] and has no expression on T cells or CD34+ hematopoietic progenitors. In a preclinical study, CD1a CAR T cells have been shown to be fratricide resistant and a safe treatment for relapsed/refractory coT-ALL patients [57,86]. CD30 is expressed by a subset of activated T cells, including almost all Hodgkin lymphomas (HL) and anaplastic large cell lymphomas (ALCL), and approximately 38% of T-ALL. Furthermore, Zheng et al. reported the expression of CD3 to be upregulated during high-dose chemotherapy in T-ALL patients [87]. Currently, 11 clinical trials concerning anti-CD30 CAR T therapy in CD30-positive malignancies are ongoing (NCT01316146, NCT01192464, NCT03049449, NCT02690545, NCT02958410, NCT02663297, NCT03383965, NCT02917083, NCT04008394, NCT02259556, and NCT03602157) and each is at a different stage of clinical trials. One of the clinical trials, involving 41 patients showed an overall response rate of 72%, a CR rate of 59%, and none of the patients suffered life-threatening infections [88]. 

Another promising approach is the incorporation of suicide genes and switches into the CAR constructs to restrict the life and activities of CAR T cells (Figure 4B). Gina Ma et al. used CAMPATH (alemtuzumab) as a natural safety switch to deplete the infused CD4 CAR T cells. This was shown to avert CAR T-caused CD4 cell aplasia after eliminating CD4-positive T-ALL in a systemic mouse model [46]. Their results demonstrated that CD4 CAR T cells have robust antitumor activities in vivo, and CAR T cells were depleted 24 h after CAMPATH treatment in different organs. In addition, bridging to an allogeneic HSCT following CAR T-cell therapy and using short-lived effector cells instead of T cells have been shown to be valid methods to prevent T-cell aplasia, with both strategies succeeding in clinical studies [89,90]. 

## 4. Product Contamination and Potential Strategies

One important issue in the field of CAR T-cell therapy is the contamination of CAR T-cell products with tumor cells, which subsequently generates CAR-modified tumor cells with immunity to CAR T cells [33]. The CAR molecule ‘masks’ the antigen on the tumor cells, preventing it from being recognized by CAR T cells, which has serious implications for manufacturing safety [91]. In 2018, Ruella et al. reported a patient relapsing after anti-CD19 CAR T-cell infusion with CD19 positive B-ALL that abnormally expressed the anti-CD19 CAR. CAR transduced B-cell leukemia (CARB) cells expanded out of control and the patient ultimately died of complications related to progressive leukemia [92].

Product contamination occurs when malignant and normal T cells are both isolated in the course of leukapheresis, especially for T-cell lymphoma or T-ALL [33]. In addition, the removal of cancer cells before gene transfer using cell purification methods can be used to address this issue. However, traditional cell purification methods such as fluorescence-activated cell separation and magnetic-activated cell separation have proven to be unsuitable for high-purity T-cell purification [93,94]. Recently, one group developed a label-free high-throughput cell purification method (dielectrophoretic cell purification) that was shown to achieve 100% purity with a high cell viability (greater than 90%). This indicates that this method is a feasible program for allogeneic CAR T therapy. However, circulating tumor T cells were often identified in the peripheral blood of T-ALL patients, which significantly increases the risk of transducing malignant cells when isolating autologous T cells [95,96,97].

**Figure 5:**
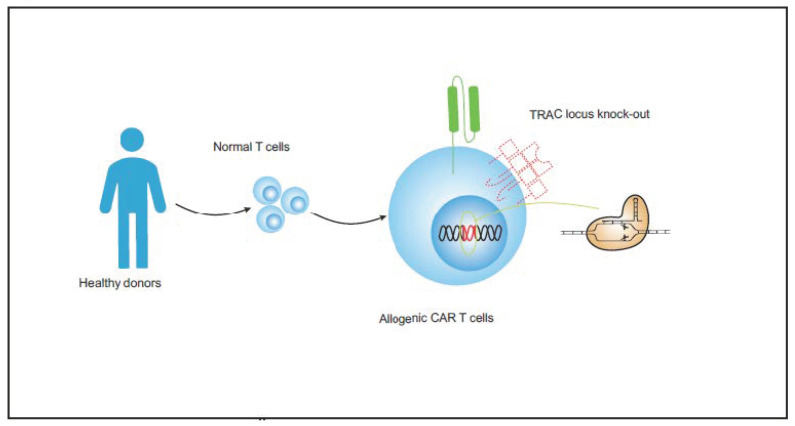
Preparation process of allogeneic CAR T cells from healthy donors.

Generating a CAR T-cell product using cells from healthy donors could be used to entirely avoid this problem and has been tested both in preclinical studies and clinical trials (Figure 5, Table 2). In addition, the risk of GvHD and host-mediated rejection should also be avoided in allogeneic CAR T-cell therapies. In 2018, Cooper et al. demonstrated “off-the-shelf” CD7 CAR T, which deleted both CD7 and T-cell receptor alpha chain (TRAC) expression using CRISPR/Cas9. This demonstrated efficacy against T-ALL without developing xenogeneic GvHD in vivo [85,98]. 

Independent antigenic presentation by MHC molecules for recognition makes γδT cells ideal candidates for allogeneic cell therapy [99,100]. Currently, preclinical studies and early-phase clinical trials have demonstrated that ex vivo expanded Vγ9Vδ2 T cells display significant antitumor activities [101,102]. Laurence JN Cooper’s group demonstrated that γδCAR T cells displayed enhanced cytotoxicity against tumor cells in vitro [103]. Furthermore, Rozenbaum et al. reported the high transduction efficacy of γδ T cells compared with standard CAR T cells (60.5 ± 13.2 and 65.3 ± 18.3%, respectively) and demonstrated that γδCAR T cells targeting CD19 could be effective against CD19+ cell lines in vitro and in vivo and could also kill CD19 antigen negative leukemia cells [104]. Since γδT cells can be safely employed in the allogeneic setting and exhibit a robust antitumor response, γδT cells may represent a better candidate for universal CAR (UCAR) T therapy than αβT.

UCAR T technology is constantly evolving and can be used to prevent adverse effects by combining multiple gene editing strategies. It should be considered as a potential therapy for clinical trials.

In addition to the other three shortcomings, CAR T-cell dysfunction is also a disadvantage of CAR T therapy. It involves T-cell exhaustion (manifested as the loss of one of the following properties: expansion, secretion of effector cytokines and lysing target cells, surviving after antigen stimulation, and performing secondary antigen challenge) and T-cell senescence, meaning that they are not able to proliferate in response to antigen stimulation [105]. Costimulatory molecules, such as OX40 and 4-1BB, may be used to enhance T-cell antitumor activity [106]. Checkpoint blockade therapy has been approved for clinical use to combat T-cell exhaustion by increasing proliferation of less differentiated T-cell populations and generating a large pool of terminally exhausted T cells, which allows for therapeutic combinations with activated costimulatory molecules and produces noteworthy effects [107,108]. Moreover, in contrast to the CD8+ subpopulation, CD4+ T cells seem to be less vulnerable to exhaustion and apoptosis and could be better used in CAR T therapy [109,110]. CAR T cells derived from post-chemotherapy patient T cells may undergo treatment-induced cellular senescence [111], which shows that immune cells should be harvested earlier in the course of patient treatment before they are significantly harmed by chemotherapy. Generally, we believe that strategies to combat T-cell dysfunction will be beneficial to CAR T therapy.

## 5. Discussion

CAR T cells represent a “living drug” that targets patients with r/r B-ALL with impressive results [112,113,114]. Many studies on the targets of immunotherapy against T-cell malignancies, especially T-ALL, are in the preclinical or early clinical research stage. The shared expression of targeted antigens between malignant and normal T cells results in fratricide, T-cell aplasia, and product contamination, which are the main drawbacks of CAR T immunotherapies for T-ALL [115]. Over recent years, several antigens with limited expression on normal T cells have been used to treat T-cell malignancies, such as TRBC1, CCR9, CD33, and CD1a, etc. [57,58,61,62]. However, most of these only express small subtypes of T-cell malignancies, which restricts these antigens in terms of a wide-ranging impact in applying CAR T therapy for patients with T-cell diseases.

**Figure 6:**
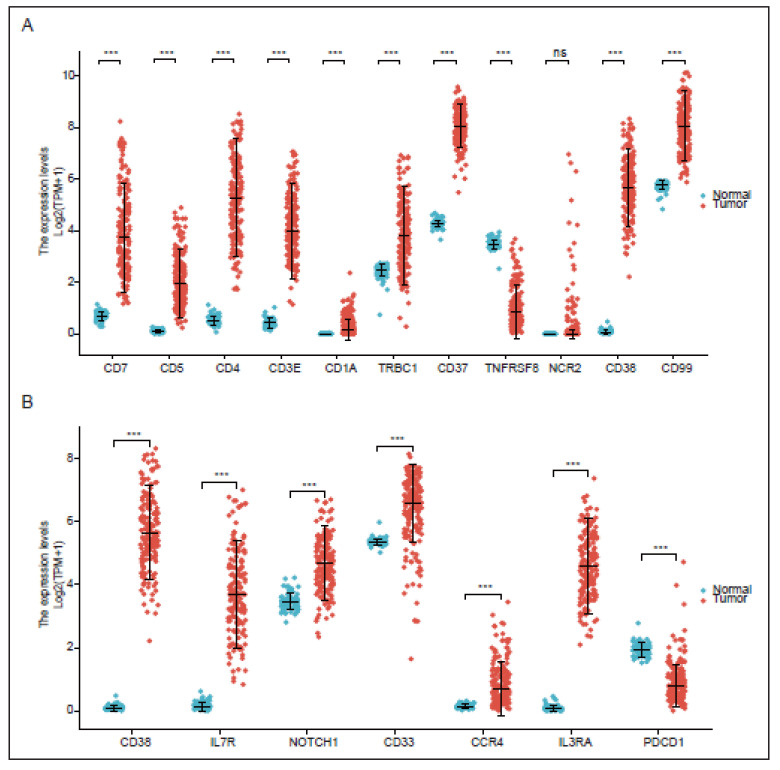
The expression of potential target antigens between tumor samples and normal samples, data from Cancer Cell Line Encyclopedia (CCLE). (**A**) The targets for CAR T therapy. (**B**) The targets for monoclonal antibody therapy. Data are mean ± SD (Two-tailed unpaired S data from Cancer Cell Line Encyclopedia (CCLE). Data are mean ± SD (Two-tailed unpaired Student’s *t* test, *** p < 0.001).

Our lab has recently reported that CD99 was specifically expressed in T-ALL and that 12E7-CD28&41-BB-based anti-CD99 CAR T expanded with minor fratricide, maintaining cytotoxic function and mediating powerful antitumor effects in vitro and in vivo. This suggests that CD99 is a promising pan-target for T-cell malignancies [59]. Nevertheless, the efficacy and safety of anti-CD99 CAR T-cell therapy are contingent on many other factors, necessitating additional research. Moreover, pan-T-cell antigens, including CD7, CD3, and CD5, were designed to treat T-cell malignancies with poor responses due to fratricide [115]. With the current development of gene editing technology, such as CRISPR-Cas9, TALEN, and CBEs technology, the problem of CAR T-cell fratricide of pan-T-cell antigens is gradually being addressed, resulting in robust clinical responses. Aside from the antigens referred to above, we also identified other genes that exhibit high expression in tumor cells that could be potential targets for CAR T therapy (Figure 6).

As a result of these issues, a second strategy involves using alternative effector cells or universal off-the-shelf T cells. These were demonstrated to be promising solutions to overcome fratricide and product contamination [44,45,116]. There are numerous ongoing clinical studies. Certain therapies have been shown to be effective and others are still under in-depth assessment.

Although CAR T cells designed for T-cell malignant tumors have various issues, the identification of more specific antigens, the advancement of gene editing technology, and the continuous in-depth research on other effector cells, such as NK cells and myeloid cells, shows that CAR T-cell therapy is a promising therapeutic opportunity for T-cell malignancies. The results from these clinical trials are eagerly awaited. 

## 6. Prospective Research Areas

The concept of a chimeric antigen receptor (CAR) was established in 1989 [117]. After more than three decades of research, CAR T therapy has yielded better results in the treatment of hematologic tumors than other existing immunotherapies. However, as a result of the characteristics of T-cell tumors, CAR T therapy still has many limitations in the treatment of T-cell malignancies. More research and trials are needed to improve clinical outcomes. Various important prospective research areas are listed below:

(1) Transducing CAR on other types of immune cells beyond T cells. NK cells, the NK-92 cell line, γδT cells, and macrophages have been used as CAR-transduced effector cells instead of T cells to treat tumors, and several clinical trials are ongoing to evaluate the effect in a variety of tumor types (NCT05007379, NCT04660929, NCT04702841). We believe that more therapy focused on applying γδT cells, macrophages, and induced pluripotent stem (iPS) cell-derived NK cells should be initiated to treat T-ALL;
(2) Identifying more specific antigens limitedly expressed on normal T cells. TRBC1/2, CDR3, CD1a, CCR7, CCR9, CD33, CD30, and CD99 have been identified and tested as targets for CAR T therapy in the treatment of T-cell malignancies. These antigens have limited expression on normal T cells or only express one subtype of tumor T cells, which allows CAR T cells and normal T cells to proliferate normally. Previous research has largely focused on single antigen CAR T therapy in T-cell malignancies; however, dual antigens are superior and thus this area requires further investigation;
(3) Optimizing cell editing technology controlling the antigens expressed on CAR T. TALEN and CRISPR/Cas9 have been used to knock out the target antigen expressed on CAR T cells, preventing CAR T-cell fratricide. Diorio et al. created cytosine base editor (CBE) technology, which was applied to create CD7-directed allogeneic CAR T using four simultaneous base edits [80]. This study suggests a promising potential method for genome editing of antigens in cellular products without the unpredictable and undesirable outcomes associated with CRISPR-cas9. Thus, the CBE technology could be used to knock out other antigens, and more optimized gene-editing techniques can be created to inhibit CAR T-cell fratricide in the future;
(4) Updating and developing the universal CAR T-cell therapy for T-cell malignancies. Allogeneic CAR T cells from healthy donors could help to prevent problems associated with poor CAR T-cell quality and product contamination. The combination of UCAR T cell and gene-editing techniques could represent a mature biological product and become the trend of future T-ALL treatments.

**Table 1:** Potential targets for immunotherapy of T-cell malignancies.

Therapy	Target	Clinical Trials	Eligible Disease	Solved Problems	References
CD7 CAR-T CD7 CAR-NK	CD7	NCT03690011 NCT04934774 NCT04840875 NCT05059912 NCT04599556 NCT04689659 NCT04762485 NCT02742727 ChiCTR190002531 ChiCTR2000034762 ChiCTR2000038714 ChiCTR2000040641 ChiCTR2100042807 ChiCTR2100043252 ChiCTR2100045863	T-ALL CD7^+^ PTCL		Png, Y.T. et al., 2017 [41], You, Y. et al., 2019 [42], Gomes-Silva, D. et al., 2017 [50], Zhang, M. et al., 2021 [81]
CD5 CAR-T CD5 CAR-NK	CD5	NCT03081910 NCT04594135 NCT05032599 ChiCTR2000039519	CD5^+^ T-ALL CD5^+^ PTCL		Chen, K.H. et al., 2017 [39], Raikar, S.S. et al., 2018 [43]
CD4 CAR-T CD4 CAR-NK	CD4	NCT03829540 NCT04162340 NCT04712864 ChiCTR2100042782	CD4^+^ T-ALL CD4^+^ PTCL	Fratricide	Pinz, K. et al., 2017 [52], Ma, G. et al., 2019 [84]
CD3 CAR-NK	CD3	N/A	T-ALL		Chen et al., 2016 [47], Rasaiyaah, J. et al., 2018 [74]
CD1a CAR-T	CD1a	N/A	Cortical T-ALL	Fratricide T-cell aplasia	Sánchez-Martínez, D. et al., 2019 [86]
AUTO (an autologous CAR-T product)	TRBC1	NCT03590574 EudraCT2017-001965-26	TRBC1^+^ T-NHL	Fratricide T-cell aplasia	Maciocia, P.M. et al., 2017 [65]
CD37 CAR-T	CD37	N/A	CD37^+^ PTCL		Scarfò et al., 2018 [118]
CDR3 CAR-T	CDR3	N/A	T-cell leukemia/lymphoma	Fratricide T-cell aplasia	Huang, J. et al., 2019 [46]
CD30 CAR-T	CD30	NCT01979536 NCT02729961 NCT02917083 NCT03383965 NCT04008394 NCT01316146 NCT01192464 NCT03049449 NCT02690545 NCT02958410 NCT02663297 NCT02259556 NCT03602157 ChiCTR-OPN-16009069 ChiCTR2000030843 ChiCTR2100046763 EudraCT2019-001263-70	ALCL ALCL CD30^+^ PTCL ALCL CD30^+^ PTCL	Fratricide T-cell aplasia	Zhang, S. et al., 2022 [72], Zheng, W. et al., 2014 [87], Ramos, C.A. et al., 2020 [88]
CD99 CAR-T	CD99	ChiCTR2000033989 ChiCTR2100046764	T-ALL	Fratricide T-cell aplasia	Shi, J. et al., 2021 [59]

**Table 2:** Clinical trials for solving product contamination.

Therapy	Target	Clinical Trials	Eligible Disease	Solved Problems
CD7 U-CAR-T CD7 Allogeneic CAR-T	CD7	NCT04264078 NCT05377827 NCT04984356 NCT05509855 NCT05127135	CD7+ T-ALL T-cell leukemia/lymphoma	Product contamination
CD5 Allogeneic CAR-T	CD5	NCT03081910	CD5+ T-ALL CD5+ T-NHLs	Product contamination
CD30 Allogeneic CAR-T	CD30	NCT04288726 NCT04952584	CD30+ T-cell lymphoma	Product contamination

The advancements in CAR T therapy over the last 30 years will pave the way for the treatment of hematopoietic malignancies and open new doors to investigating novel therapeutic strategies for treating cancers.
